# Demographic Profile of Non-Odontogenic Jaw Lesions in an Iranian Population: A 30-Year Archive Review

**Published:** 2017-05

**Authors:** Fereshteh Baghai Naini, Pouyan Aminishakib, Maedeh Ghorbanpour, Mohammad Mehdi Vakili, Mohammad Javad Kharazifard

**Affiliations:** 1 Associate Professor, Dental Research Center, Dentistry Research Institute, Tehran University of Medical Sciences, Tehran, Iran; Department of Oral and Maxillofacial Pathology, School of Dentistry, Tehran University of Medical Sciences, Tehran, Iran; 2 Assistant Professor, Department of Oral and Maxillofacial Pathology, School of Dentistry, Tehran University of Medical Sciences, Tehran, Iran; 3 Adjunct Assistant Professor, Department of Oral and Maxillofacial Pathology, School of Dentistry, Tehran University of Medical Sciences, Tehran, Iran; 4 Dentist, Private Practice, Tehran, Iran; 5 Epidemiologist, Dental Research Center, Dentistry Research Institute, Tehran University of Medical Sciences, Tehran, Iran

**Keywords:** Prevalence, Jaw, Nonodontogenic Cysts, Retrospective Studies

## Abstract

**Objectives:**

The frequency of non-odontogenic lesions of the jawbones is lower than that of odontogenic lesions; however, study of the epidemiologic data of these lesions is required for health care programs. This study aimed to assess the relative frequency and demographic profile of non-odontogenic jaw lesions in an Iranian population over a 30-year period.

**Materials and Methods:**

This archive review was performed using demographic and biopsy information of all patients with non-odontogenic lesions of the jawbones submitted to the Oral Pathology Department of Dental School of Tehran University of Medical Sciences from 1984 to 2014. Demographic data included in the study were: age at diagnosis, gender and location of lesion. The lesions were divided into three groups of group 1: cystic lesions, group 2: tumors and tumor-like lesions, and group 3: infectious/inflammatory/reactive lesions. Frequency and clinical data were analyzed using SPSS 22.

**Results:**

Of 972 non-odontogenic jaw lesions, the ratio of mandibular to maxillary lesions was 1.63:1. Female to male ratio was 1.33:1 and the mean age of patients was 29.09±16.90 years. The most common non-odontogenic jaw lesion was central giant cell granuloma (CGCG). In groups 1, 2 and 3, nasopalatine duct cyst, CGCG, and osteomyelitis were the most frequent lesions, respectively.

**Conclusions:**

Non-odontogenic lesions of the jawbones are a diverse group of lesions with different frequency and behavior. This study demonstrated that tumors and tumor-like lesions of the jaws were more common than cystic and infectious/inflammatory/reactive lesions. Overall, the most common non-odontogenic jaw lesion was CGCG.

## INTRODUCTION

The jaws may be affected by a wide variety of lesions consisting of odontogenic and non-odontogenic lesions. Odontogenic lesions, comprising of cysts and tumors, are the lesions arising from the odontogenic apparatus [1]. Several studies from around the world have reported the relative frequency of odontogenic lesions [2–10], including studies from Iran [11–14]. Likewise, the epidemiological profile for non-odontogenic lesions has been studied previously in different populations [2, 3, 6, 15, 16], but few studies in Iran have addressed this issue. In general, the frequency of non-odontogenic lesions of jawbones is less than that of odontogenic lesions [17]; however, study of the epidemiologic data of these lesions for accurate diagnosis of each entity is mandatory, because treatment and prognosis of variable lesions are different. Similarly, health systems in each country require precise information regarding disease occurrence to make regulatory decisions, rules and policies for health planning and to effectively allocate resources [2]. Since geographic distribution is a source of variation, it seems logical to study this topic in our country. The best source to obtain such information is the records of oral pathology diagnostic services. Information gained from these archives, especially from large centers of this field is valuable and probably represents the larger community [8]. Therefore, the purpose of this study was to assess the relative frequency and demographic profile of non-odontogenic jaw lesions in an Iranian population over a 30-year period.

## MATERIALS AND METHODS

This archive review was performed using the demographic and biopsy information of all patients with oral intra-osseous lesions submitted to the Department of Oral and Maxillofacial Pathology of Tehran University of Medical Sciences, for a period of 30 years from 1984 to 2014. Selection of intra-osseous lesions was according to the previous histopathologic diagnosis of the lesions, and specimens with uncertain diagnosis or with incomplete information were excluded from the study. The demographic data included in the study were: age at the time of diagnosis, gender and location of lesion. The next step was separating the odontogenic lesions from non-odontogenic ones according to the latest edition of Neville oral and maxillofacial pathology textbook [18]. Since the name of some entities had changed over time, and thus they had been recorded by different names, the same lesions with different names were reclassified and renamed according to the textbook. Because of the lack of sufficient clinical history and radiographic data for some of the fibro-osseous lesions, we did not classify these lesions and we used the general term of fibro-osseous lesions. Then, the lesions were divided into three groups of group 1: cystic lesions, group 2: tumors and tumor-like lesions, and group 3: infectious/inflammatory/reactive lesions. Frequency and clinical data were analyzed using SPSS 22 (SPSS Inc., IL, USA).

## RESULTS

Out of 3,669 intra-osseous jaw lesions found during the 30-year period, 2,697 (73.5%) were odontogenic and 972 (26.4%) were non-odontogenic making odontogenic lesions 2.77 times more common than non-odontogenic lesions. Of non-odontogenic lesions, 346 (35.6%) involved the maxilla, 566 (58.2%) involved the mandible, 3 (0.3) involved both jaws, and location of 57 (5.8%) cases was not specified. The ratio of mandibular to maxillary lesions was 1.63:1. Of these, 549 (56.4%) cases were in females, 412 (42.3%) cases were in males, and the gender of 11 (1.1%) cases was not specified. The age range of these patients was 2–90 years with a mean age of 29.09±16.90 years. The most frequent nonodontogenic lesion was central giant cell granuloma (CGCG) (n= 341; 35%), followed by ossifying fibroma (n=95; 9.7%), osteomyelitis (n=83; 8.5%), aneurysmal bone cyst (n=53; 5.4%), fibro-osseous lesions (n=51; 5.2%), nasopalatine duct cyst (n=48; 4.9%), Langerhans cell histiocytosis (n=34; 3.5%), lymphoma (n=27; 2.7%), osteoma (n=27; 2.7%), fibrous dysplasia (n=25; 2.5%), osteosarcoma (n=25; 2.5%) and traumatic bone cyst (n=25; 2.5%). In this study, tumors and tumor-like lesions (n=779; 80.1%) were more frequently seen than cystic (n=64; 6.6%) and infectious/inflammatory/reactive lesions (n=129; 13.3%). Table 1 shows the frequency and demographic data of each group. In group 1, nasopalatine duct cyst was the most common lesion observed (n=48). This lesion was frequently seen in males and the mean age of patients was 39.56±16.10 years. Table 2 demonstrates the frequency and demographic data of patients with cystic lesions. In group 2, CGCG was the most frequent lesion identified (n=341). This lesion was frequently seen in the mandible and females, and the mean age of patients was 25.47±15.56 years. Table 3 demonstrates the frequency and demographic data of patients with tumors and tumor-like lesions.

**Table 1:** Frequency and demographic data of each group of lesions

**Group**	**Number (%)**	**Gender**			**Location**			

		**NS (%)**	**Female (%)**	**Male (%)**	**Max. (%)**	**Man. (%)**	**Both (%)**	**NS (%)**
**Cystic lesions**	64 (6.6)	0 (0.0)	24 (37.5)	40 (62.5)	64 (100.0)	0 (0.0)	0 (0.0)	0 (0.0)
**Tumors and tumor-like lesions**	779 (80.1)	9 (1.1)	453 (58.1)	317 (40.7)	252 (32.3)	474 (60.8)	3 (0.3)	50 (6.4)
**Infectious/inflammatory/reactive lesions**	129 (13.3)	2 (1.5)	72 (55.8)	55 (42.6)	31 (24.03)	92 (71.3)	0(0.0)	6 (4.6)
**Total**	972 (100.0)	11 (1.1)	549 (56.4)	412 (42.3)	346 (35.6)	566 (58.2)	3 (0.3)	57 (5.8)

NS= Not specified; Max.= Maxilla; Man.= Mandible

**Table 2:** Frequency of cystic lesions and their distribution according to gender, age and location

**Lesion**	**Number (%)**	**Gender**			**Mean age**	**Location**			
	
		**NS (%)**	**Female (%)**	**Male (%)**		**Max.(%)**	**Man. (%)**	**Both (%)**	**NS (%)**
**Nasopalatine duct cyst**	48 (75.0)	0 (0.0)	18 (37.5)	30 (62.5)	39.5	48 (100.0)	0 (0.0)	0 (0.0)	0 (0.0)
**Surgical cilliated cyst**	16 (25.0)	0 (0.0)	6 (37.5)	10 (62.5)	44.9	16 (100.0)	0 (0.0)	0 (0.0)	0 (0.0)
**Total**	64 (100.0)	0 (0.0)	24 (37.5)	40 (62.5)		64 (100.0)	0 (0.0)	0 (0.0)	0 (0.0)

NS= Not specified; Max.= Maxilla; Man.= Mandible

**Table 3:** Frequency of tumors and tumor-like lesions and their distribution according to gender, age and location

**Lesion**	**Number (%)**	**Gender**			**Mean age**	**Location**			
	
		NS (%)	Female (%)	Male (%)		Max. (%)	Man. (%)	Both (%)	NS (%)
**CGCG**	341 (43.7)	7 (2.05)	216 (63.3)	118 (34.6)	25.47	107 (31.3)	216 (63.3)	0 (0.0)	18 (5.2)
**Ossifying fibroma**	95 (12.2)	0 (0.0)	59 (62.1)	36 (37.8)	29.16	23 (24.2)	63 (66.3)	0 (0.0)	9 (9.4)
**Aneurysmal bone cyst**	53 (6.8)	0 (0.0)	31 (58.5)	22 (41.5)	22.36	12 (22.6)	39 (73.5)	0 (0.0)	2 (3.77)
**Fibro-osseous lesion**	51 (6.5)	1 (1.9)	40 (78.4)	10 (19.6)	31.16	16 (31.3)	31 (60.7)	1 (1.9)	3 (5.8)
**Langerhans cell histiocytosis**	34 (4.3)	0 (0.0)	9 (26.4)	25 (73.5)	18.96	11 (32.3)	18 (52.9)	0 (0.0)	5 (14.7)
**Lymphoma**	27 (3.4)	0 (0.0)	6 (22.2)	21 (77.7)	35.98	17 (62.9)	9 (33.3)	0 (0.0)	1 (3.7)
**Osteoma**	27 (3.4)	0 (0.0)	16 (59.2)	11 (40.7)	33.56	11 (40.7)	11 (40.7)	0 (0.0)	5 (18.5)
**Fibrous dysplasia**	25 (3.2)	1 (4.0)	12 (48.0)	12 (48.0)	25.30	17 (68.0)	6 (24.0)	0 (0.0)	2 (8.0)
**Osteosarcoma**	25 (3.2)	0 (0.0)	10 (40.0)	15 (60.0)	28.66	9 (36.0)	16 (64.0)	0 (0.0)	0 (0.0)
**Cemento-osseous dysplasia**	11 (1.4)	0 (0.0)	10 (90.9)	1 (9.1)	41.33	2 (18.1)	9 (81.8)	0 (0.0)	0 (0.0)
**Spindle cell tumor**	10 (1.2)	0 (0.0)	8 (80.0)	2 (20.0)	30.9	0 (0.0)	9 (90.0)	0 (0.0)	1 (10.0)
Other tumors & tumor-like lesions^*^	80 (10.2)	0 (0.0)	36 (45.0)	44 (55.0)		27 (33.75)	47 (58.75)	2 (2.5)	4 (5.0)
**Total**	779 (100.0)	9 (1.1)	453 (58.1)	317 (40.7)		252 (32.3)	474 (60.8)	3 (0.3)	50 (6.4)

**CGCG: Central giant cell granuloma; NS= not specified; Max.= Maxilla; Man.= Mandible**

* **Cherubism (n=9); small round cell sarcoma (n=9); spindle cell sarcoma (n=9); chondrosarcoma (n= 8); plasmacytoma (n=7); central mucoepidermoid carcinoma (n=4); ewing’s sarcoma (n=4); juvenile ossifying fibroma (n=4); osteoblastoma (n=3); adenocarcinoma (n=2); cavernous hemangioma (n=2); desmoplastic fibroma (n=2); intraosseous squamous cell carcinoma (n=2); metastatic carcinoma (n= 2); neurofibroma (n= 2); osteoid osteoma (n= 2); benign myxoid tumor (n=1); chondroblastoma (n=1); chondroma (n=1); gigantiform cementoma (n=1); hemangioma (n=1); high grade spindle cell sarcoma (n=1); mature teratoma (n=1); osteochondroma (n=1); solitary plasmacytoma (n=1)**

In group 3, osteomyelitis was the most common lesion observed (n=83) This lesion was frequently seen in the mandible and females and the mean age of patients was 38.23±15.99 years. Table 4 demonstrates the frequency and demographic data of patients with infectious/inflammatory/reactive lesions.

**Table 4:** Frequency of infectious/inflammatory/reactive lesions and their distribution according to gender, age and location

**Lesion**	**Number (%)**	**Gender**			**Mean age**	**Location**			
	
		NS (%)	Female (%)	Male (%)		Max. (%)	Man. (%)	Both (%)	NS (%)
**Osteomyelitis**	83 (64.3)	1 (1.20)	39 (46.9)	43 (51.8)	38.2	24 (28.9)	55 (66.2)	0 (0.0)	4 (4.8)
**Traumatic bone cyst**	25 (19.3)	1 (4.0)	20 (80.0)	4 (16)	20.69	0 (0.0)	24 (96.0)	0 (0.0)	1 (4.0)
**Condensing osteitis**	7 (5.4)	0 (0.0)	4 (57.1)	3 (42.8)	33.66	3 (42.8)	4 (57.1)	0 (0.0)	0 (0.0)
**Idiopathic osteosclerosis**	6 (4.6)	0 (0.0)	4 (66.6)	2 (33.3)	37.83	1 (16.6)	5 (83.3)	0 (0.0)	0 (0.0)
**Focal Osteoporotic marrow defect**	4 (3.1)	0 (0.0)	3 (75.0)	1 (25.0)	47.25	1 (25.0)	2 (50.0)	0 (0.0)	1 (25.0)
**Chronic actinomycotic abscess**	2 (1.5)	0 (0.0)	2 (100.0)	0 (0.0)	20.00	2 (100.0)	0 (0.0)	0 (0.0)	0 (0.0)
**Massive osteolysis**	1 (0.7)	0 (0.0)	0 (0.0)	1 (100.0)	14.00	0 (0.0)	1 (100.0)	0 (0.0)	0 (0.0)
**Osteonecrosis**	1 (0.7)	0 (0.0)	0 (0.0)	1 (100.0)	43.00	0 (0.0)	1 (100.0)	0 (0.0)	0 (0.0)
**Total**	129 (100.0)	2 (1.5)	72 (55.8)	55 (42.6)		31 24.03)	92 (71.3)	0 (0.0)	6 (4.6)

NS= Not specified; Max.= Maxilla; Man.= Mandible

## DISCUSSION

Epidemiologic studies are known as the basis for health policy making. Geographic distribution of diseases and the influence of ethnic, nutritional and cultural habits, and genetic patterns, have great importance in occurrence and prevalence of a particular disease. For instance, a disease which is prevalent in a particular community, may be seen rarely in another community. Therefore, studying the epidemiological profile of various diseases is important to identify the etiology and risk factors of the most common diseases, to establish new prevention and treatment protocols, making regulatory decisions, rules and policies for health planning, and to effectively allocate resources [19].

Non-odontogenic lesions of the jawbones are a diverse group of lesions with different frequency and behavior. Few studies in Iran have addressed this topic. It was somewhat difficult to compare the results of this study with those of other studies, because some of the previous studies were performed on both odontogenic and nonodontogenic jaw lesions [2, 17, 20], and some of them investigated a specific group for example oral non-odontogenic cysts [4, 9, 15, 21] or tumors [6, 22]. In addition, some of the studies evaluated the prevalence of all oral lesions in a specific population [3, 23, 24] rather than investigating only the intraosseous jaw lesions.

Regarding the 30-year period of evaluation and the number of non-odontogenic cases investigated, the present study is the first in Iran. This retrospective study was performed using the demographic and biopsy information of all patients with nonodontogenic lesions of the jaws submitted to the Department of Oral and Maxillofacial Pathology of Tehran University of Medical Sciences, Iran, Tehran, from 1984 to 2014. Out of 3,669 intraosseous jaw lesions accessed, 2,697 (73.5%) cases were odontogenic and 972 (26.4%) cases were nonodontogenic. In the present study, the occurrence rate of non-odontogenic lesions in the mandible was greater than in the maxilla, which is in accordance with the findings of Ali [20] in Kuwait, Johnson et al, [2] in Australia, and Tatli et al, [25] in Turkey. However, the exact location of the lesions in the maxilla or mandible (posterior or anterior) was not recorded for some cases in our archive, and therefore this was one of the limitations of our study. Our findings demonstrated increased propensity for these lesions to occur in females (n=549; 56.4%), which is consistent with the findings of Ali [20] in Kuwait and Johnson et al, [2] in Australia. Tatli et al. [25] reported greater prevalence of nonodontogenic cysts in males and non-odontogenic tumors in females, with overall predominance in females. The age range of patients was 2–90 years with a mean age of 29.09±16.90 years; however, in a study in Australia, the age range of patients was 7–81 years with a mean age of 38.6 [2]. In the current study, the most common non-odontogenic jaw lesion was CGCG (35% of the cases). Siadati et al, [3] in Iran also reported similar findings (CGCG was the commonest lesion among the non-odontogenic intraosseous lesions that they reported), but these findings were inconsistent with the results of Johnson et al, [2] in Australia and Ali [20] in Kuwait, who reported the nasopalatine duct cyst to be the commonest non-odontogenic jaw lesion. A probable explanation for this difference could be related to ethnic or geographic variations. In the present study, tumors and tumor-like lesions of the jaws (group 2) were the most common among the three groups (n=779; 80.1%), which is in contrast to the results of Ali [20] in Kuwait, who reported that the cystic lesions occurred more frequently than tumors. This difference might be due to the high number of cases that we evaluated through the 30-year period. The most common non-odontogenic cyst was nasopalatine duct cyst in our study, which is consistent with the findings of previous reports from Australia, Turkey, Canada, Iran, Kuwait, Brazil, United Kingdom, and Kenya [2, 4, 9, 15, 17, 20, 21, 23, 26]. The most common non-odontogenic tumor or tumor-like lesion in the present study was CGCG, which is in agreement with the results of previous reports from Iran, Libya, Kuwait, and Brazil [3, 6, 20, 24]. Johnson et al, [2] in Australia reported fibrous dysplasia and CGCG to be the most frequent non-odontogenic tumor or tumor-like lesion. Osteomyelitis was among the most common infectious/inflammatory/reactive processes in our study. Ali [20] in Kuwait reported chronic apical periodontitis to be the most common inflammatory lesion, which has an odontogenic origin, followed by osteomyelitis. Therefore, this finding is in line with the results of Ali [20].

## CONCLUSION

Non-odontogenic lesions of the jawbones are a diverse group of lesions with different frequency and behavior. This study demonstrated that tumors and tumor-like lesions of the jaws were more common than cystic and infectious/inflammatory / reactive lesions. Overall, the most common nonodontogenic jaw lesion in Iran has been CGCG. The most common non-odontogenic tumor or tumor-like lesion in the present study was also CGCG. Among the cystic lesions, nasopalatine duct cyst and among the infectious/ inflammatory/ reactive lesions, osteomyelitis was the most frequent lesion encountered.
